# Observation of the Charge Resonance Band of Hemibonded (H_2_O)_2_^+^ in the Gas Phase

**DOI:** 10.1021/acs.jpclett.6c01649

**Published:** 2026-06-19

**Authors:** Mizuhiro Kominato, Tatsuki Hosoda, Kenta Mizuse, Susumu Kuma, Andrey F. Vilesov, Asuka Fujii

**Affiliations:** † Department of Chemistry, Graduate School of Science, 13101 Tohoku University, Sendai 980-8578, Japan; ‡ Department of Chemistry, School of Science, Kitasato University, Sagamihara, Kanagawa 252-0373, Japan; § Department of Physics, College of Science, 13024 Rikkyo University, Toshima 171-8501, Japan; ∥ Department of Chemistry and Department of Physics and Astronomy, 5116 University of Southern California, Los Angeles, California 90089, United States

## Abstract

Electronic spectroscopy of (H_2_O)_*n*_^+^ (*n* = 2 and 3) radical cluster cations was investigated in the ultraviolet–visible region. For (H_2_O)_2_^+^, the charge resonance band attributable to the hemibonded isomer, an elusive species previously observed spectroscopically only in helium droplets, was identified in the gas phase. This observation establishes electronic spectroscopy as a sensitive probe of hemibonded structures in water radical cations.

## Graphical abstract

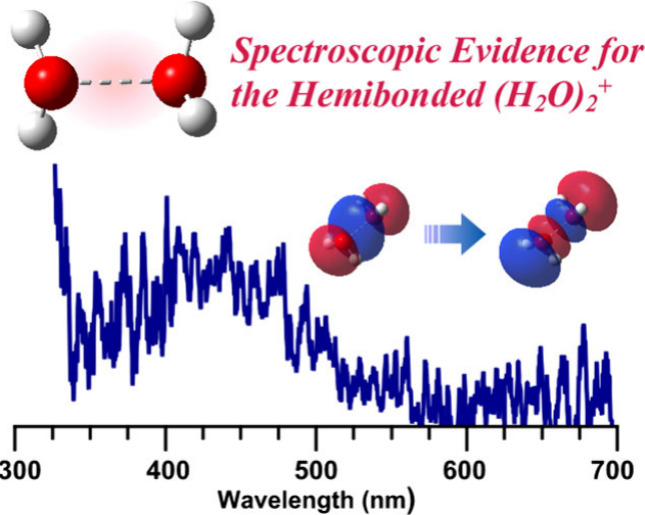

The ionization dynamics of water have attracted considerable interest and have been extensively investigated.^1–56^ In particular, recent advances in ultrafast spectroscopic techniques elucidated the transient phenomena occurring on the extremely short timescale immediately following the ionization of liquid water.^2–10^

The ionization of water forms H_2_O^+^, which is unstable in an aqueous environment. It rapidly undergoes proton transfer to a neighboring hydrogen-bonded water molecule, producing protonated water (H_3_O^+^ or H_5_O_2_^+^) and an OH radical. Simultaneously, the ejected electron is captured by surrounding water molecules, becoming a solvated electron. These OH radicals and solvated electrons play central roles in the subsequent reactions. While the major processes following ionization, including proton transfer and the generation of OH radicals, hydronium ions, and solvated electrons, are well-established, important questions remain unresolved. One such question involves the participation of hemibonded water.^11–56^

A hemibond is a nonclassical covalent bond formed by the interaction of a nonbonding orbital containing a hole created by ionization with another fully occupied nonbonding orbital. The overlap of these two orbitals creates bonding (σ) and antibonding (σ*) molecular orbitals. Occupation of these orbitals by three electrons results in an effective bond with a bond order of 1/2, hence the term “hemibond” (also known as a two-center, three-electron bond or 2c-3e bond). Hemibonds are frequently observed in radical cations of third-period elements (especially sulfur).^57–60^ However, for second-period elements containing oxygen, their stability is reduced, and proton transfer strongly competes whenever it is feasible. The formation of hemibonds between water molecules has been a long-standing topic of interest, with many studies focusing on gas-phase (isolated) (H_2_O)_*n*_^+^ radical cluster cations.^11–41,46–49^ Quantum chemical calculations have played a pivotal role in understanding hemibonds of (H_2_O)_*n*_^+^.^11–34^ For instance, in the extensively studied (H_2_O)_2_^+^ system, high-level calculations indicated that the hemibonded dimer radical cation (H_2_O∴OH_2_)^+^ is a stable minimum, though the proton-transferred H_3_O^+^·OH is predicted to be 49 kJ/mol more stable (at the CCSD­(T)/CBS level).^14^ Mass spectrometry experiments have suggested the formation of hemibonded (H_2_O)_*n*_^+^,^35–41^ but obtaining direct spectroscopic evidence of the hemibonded isomer has proven extremely challenging. Infrared spectroscopic studies of (H_2_O)_*n*_^+^ generated in a supersonic jet have only yielded spectra corresponding to the proton-transferred form, with no trace of the hemibonded isomer.^46–48^ This is interpreted as being due to the facile isomerization to the more stable proton-transferred form during the collisional cooling, aided by excess energy from ionization. However, very recently, the infrared spectrum of hemibonded (H_2_O)_2_^+^ was observed in helium droplets, which provide an extremely rapid cooling.^49^ This observation marked the first definitive proof of water–water hemibond formation.

While infrared spectroscopy is highly effective for determining intermolecular structures, it is largely ineffective for detecting hemibonded water formed during the ionization of liquid water because the corresponding absorption bands of hemibonded water would overlap with the strong absorptions of neutral water.

On the other hand, hemibonds exhibit strong, structureless absorption in the ultraviolet–visible (UV–vis) region.^55,57,61–65^ This feature is referred to as a charge resonance (CR) band, based on the (σ, σ*) transition. In this spectral region, water itself does not absorb, and OH radicals formed by proton transfer competing with hemibond formation absorb around 300 nm.^66^ In contrast, the (σ, σ*) transition of the hemibond is predicted to be 1 to 2 orders of magnitude stronger than that of the OH radical, as will be shown later. Therefore, even if the proton-transferred form predominates, observing the CR band in the UV–vis region could enable the detection of even a very minor population of the hemibonded form.

In this study, we report the observation of the CR band arising from hemibonding in the water radical cation dimer (H_2_O)_2_^+^, generated in a supersonic jet. This constitutes the first observation of a CR band originating from a hemibond formed between water molecules.

Water radical cation clusters, (H_2_O)_*n*_^+^, were generated by electron impact ionization of a supersonic jet expanded from a high-pressure pulsed nozzle. The size-selected radical cations were studied using a tandem quadrupole mass spectrometer, and their UV–vis spectra were recorded via photodissociation spectroscopy. Details of the experimental procedures are described in the Supporting Information (SI).

Figure 1(a) shows the experimental UV–vis photodissociation spectrum of (H_2_O)_2_^+^. This spectrum was obtained by simultaneously monitoring both H_3_O^+^ and H_2_O^+^ fragment channels. This was necessary because, at wavelengths longer than 320 nm, the signals were too weak for spectral measurements under conditions providing sufficient mass resolution to fully separate the two fragments. In the experimental spectrum, a broad and structureless band with a maximum at 430 nm is observed, while a much more intense band rises from around 320 nm toward shorter wavelengths. As shown in Figure S1 of the SI, the latter band is found to peak at approximately 300 nm in a spectrum recorded with much weaker laser power and plotted on a reduced scale. This peak position closely matches that of the OH radical (306.4 nm in the gas phase),^66^ allowing it to be assigned to the OH moiety within the proton-transferred form of (H_2_O)_2_^+^. On the other hand, the broad band observed in the visible region cannot be explained by any transitions expected for the proton-transferred form; therefore, it is attributed to the CR band of the hemibonded form. The structureless nature of the band is also consistent with the characteristics of the CR band, which arises from a (σ, σ*) transition.^55,57,61–65^

**Figure 1:**
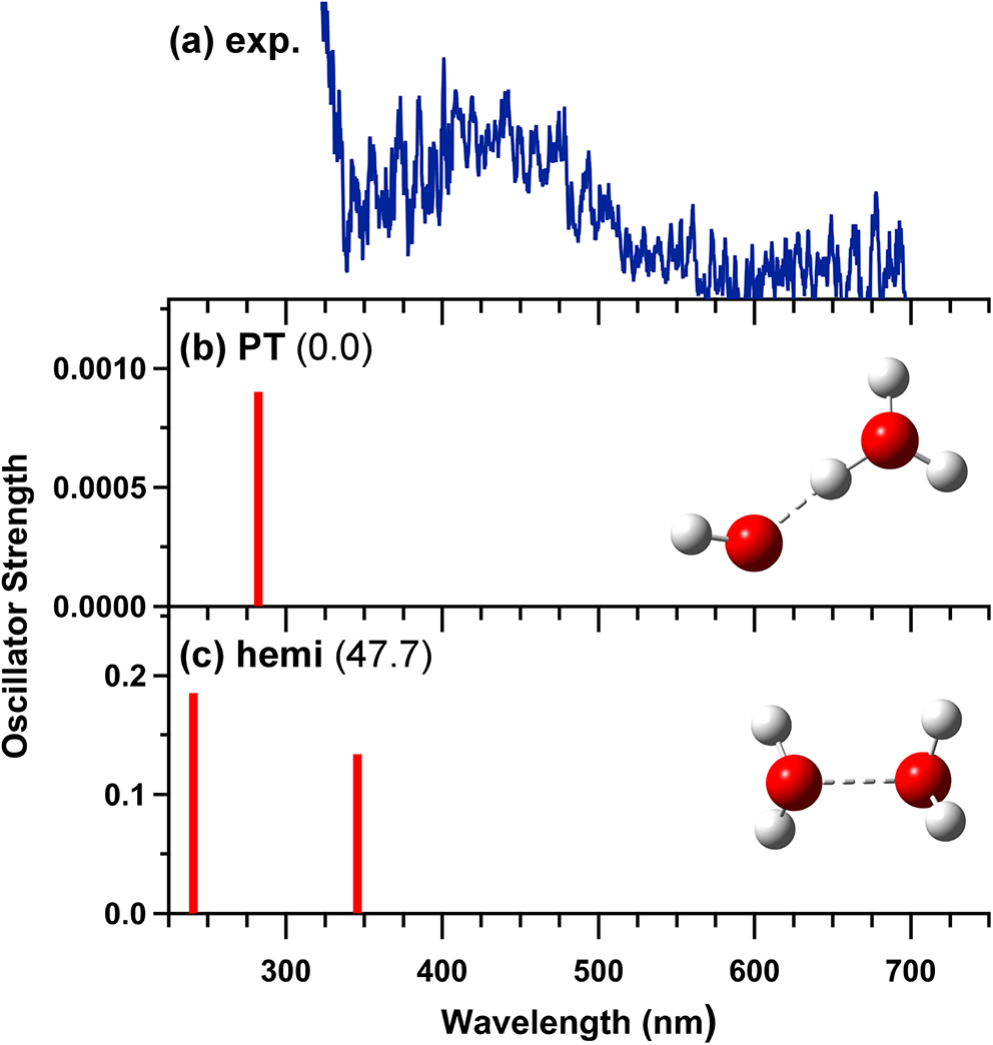
(a) UV–vis photodissociation spectrum of (H_2_O)_2_^+^ measured by monitoring the H_2_O^+^/H_3_O^+^ fragment channels. (b, c) Optimized structures and their calculated spectra of (b) the proton-transferred (PT) and (c) hemibonded (hemi) forms of (H_2_O)_2_^+^, respectively. Spectral calculations were performed at the EOM-CCSD/aug-cc-pVTZ level. The values in parentheses are the relative energies (kJ/mol) at the CCSD/aug-cc-pVTZ level, with respect to the most stable PT isomer. Zero-point energy (ZPE) corrections are included.

To ensure a more reliable assignment of the experimental spectrum, structural optimizations of (H_2_O)_2_^+^ were performed, and electronic transitions were calculated at the EOM-CCSD/aug-cc-pVTZ level based on the optimized geometries. These results are presented in Figure 1(b),(c) for the proton-transferred (PT) and hemibonded (hemi) forms, respectively. The isomeric structures and relative energies are essentially consistent with previous reports;^14,17,18,23–25,27^ ^,^ ^32–34,46,49^ the PT form is the most stable, and the hemi form is 47.7 kJ/mol higher in the present computations. In the calculated spectrum of the hemi form, a band due to the CR transition is predicted at 345 nm. Previous calculations for hemibonded (H_2_O–Ar)^+^ at the same level of theory have shown that, although this method provides the best agreement with experiment, it tends to predict band positions shifted by approximately 40 nm toward shorter wavelengths.^55^ Taking this tendency into account, the CR band of the hemi form is consistent with the observed band peaking at 430 nm. Consequently, we conclude that this observed band can be assigned to the CR band of the hemi form.

The broad, structureless nature of the observed band is consistent with a CR transition arising from a hemibond. While this bound-to-continuum transition is naturally featureless, its profile is expected to be modulated by vibronic contributions, particularly vibrational excitations along the intermolecular hemibond dissociation coordinate. However, simulating such a profile is computationally demanding, requiring full-dimensional excited-state potentials for an open-shell system and accurate temperature estimates for hot-band analysis. Therefore, further simulation of the band shape remains beyond the scope of the present work.

Meanwhile, for the most stable PT form, a transition originating from the OH moiety is predicted at 280 nm. This is in good agreement with the observed absorption onset near 320 nm; the full spectrum shown in the SI indicates that this band peaks around 300 nm. This observation is consistent with the 0–0 band of the A^2^Σ^+^-X^2^Π transition of the OH radical in the gas phase, which appears at 306.4 nm.^66^

Figure 2 shows the molecular orbitals of (H_2_O)_2_^+^ relevant to the main electronic transitions in the observed region. Their calculated transition wavelengths are summarized in Table 1. In the hemi form, the 9β and 10β orbitals are clearly identified as the σ and σ* orbitals of the hemibond formed between the two water molecules. The 8β orbital appears to be a mixture of σ and *n* orbitals. The transition calculated at 345 nm mainly consists of the 9β → 10β excitation, with an additional contribution from the 8β → 10β excitation, and thus has essentially CR character, which is consistent with the present observation. The transition calculated at 240 nm mainly consists of the 8β → 10β excitation, with an additional contribution from the 9β → 10β excitation, and may also exhibit CR band character. On the other hand, the PT form shows a transition at 280 nm, corresponding to the A^2^Σ^+^-X^2^Π transition of the OH radical.

**Figure 2:**
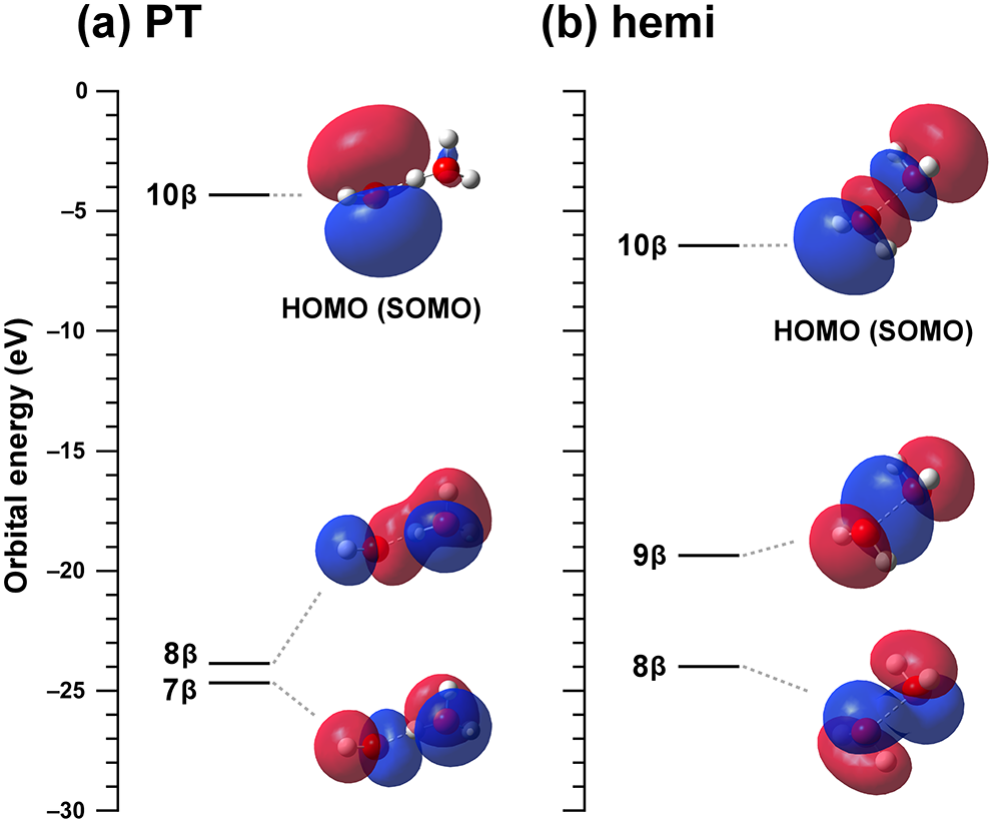
Molecular orbitals of (H_2_O)_2_^+^ relevant to the main electronic transitions and their orbital energies (in eV) for (a) the PT form and (b) the hemi form isomer. The excitation energies and their main transitions are summarized in Table 1. All calculations were performed at the EOM-CCSD/aug-cc-pVTZ level, and the molecular orbitals are plotted with an isovalue of 0.02.

**Table 1:** Transition Wavelengths, Oscillator Strengths, and Main Electronic Transitions for Each Isomer of (H_2_O)_2_^+^ ^a^

Isomer	Transition wavelength (nm)	Oscillator strength	Main transition
PT	282.16	0.0009	7β → 10β (−0.548)
			8β → 10β (0.686)
hemi	345.52	0.1340	8β → 10β (0.568)
			9β → 10β (−0.755)
	240.51	0.1850	8β → 10β (0.755)
			9β → 10β (0.563)

a Only transitions with significant excitation amplitudes are listed, and their amplitudes are given in parentheses. All calculations were performed at the EOM-CCSD/aug-cc-pVTZ level.

Notably, the transition intensity of the CR band in the hemi form is approximately 100 times stronger than that of the OH absorption in the PT form. This high intensity likely accounts for the detection of the hemi form’s signal, which has remained undetectable in previous infrared spectroscopic studies using the same ion source.^47,48^ Considering the relative transition and signal intensities, the population of the hemi form is estimated to be on the order of 10^–4^ relative to that of the PT form. However, as shown in the calculated results in Table S1 of the SI, the Gibbs energy of the hemi form is approximately 50 kJ/mol higher than that of the PT form, regardless of the temperature. Assuming thermal equilibrium between the two isomers, the population of the hemi form would be on the order of 10^–9^ that of the PT form (at 298 K, and even lower at reduced temperatures). This is clearly inconsistent with the above estimation. In the present ion production, a high-pressure supersonic jet valve (Even-Lavie valve) was employed.^67^ It has been frequently observed that its superior cooling effect leads to the kinetic trapping of high-energy isomers.^68–70^ We conclude that this effect enabled the capture of a very small population remaining in the local potential minimum, rather than relaxing to thermal equilibrium.

A similar investigation was carried out for (H_2_O)_3_^+^. The comparison between the experimental UV–vis spectra of (H_2_O)_3_^+^ and the calculated spectra of its stable isomers is provided in Figures S2 and S3 of the SI. Similar to (H_2_O)_2_^+^, a weak and featureless absorption extending from the UV into the visible region is observed, in addition to the intense OH radical absorption near 300 nm. However, unlike (H_2_O)_2_^+^, no distinct absorption maximum is visible. Furthermore, the contribution of nonhemibonded forms—which also exhibit absorption in the same region—cannot be excluded, and the relative energies of the hemibonded forms become even higher. Therefore, no definitive conclusion can be drawn regarding the observation of the CR band originating from the hemibonded form in (H_2_O)_3_^+^.

In this study, we observed transitions assigned to the CR band of the hemibonded water dimer radical cation in the near-UV to visible region. This represents the first observation of a CR band resulting from hemibonding between water molecules and the first spectroscopic confirmation of hemibonded water clusters outside of He droplets.^49^ We are planning measurements on (H_2_O)_*n*_^+^ in He droplets to observe the CR band for further confirmation. Compared to infrared spectroscopy, UV–vis (electronic) spectroscopy suffers less from interference by neutral water, making it a far more practical method for detecting hemibonded water in condensed phases. In fact, in the ultrafast transient absorption of liquid water following ionization, the 400–500 nm range lies in a gap between the strong absorptions of solvated electrons centered around 700 nm and those of OH radicals and water itself below 300 nm.^2,3,71–74^ While a weak transient absorption is known to exist in this region, its assignment has yet to be definitively established.^72–74^ We expect that this report will encourage further attempts to observe hemibonding between water molecules in condensed media.

## Supplementary Material



## Data Availability

The data supporting this article are included in the Supporting Information.

## Notes

Experimental and computational methods; experimental electronic spectrum of (H_2_O)_2_^+^ on a reduced intensity scale using attenuated laser power; experimental and calculated electronic spectra of (H_2_O)_3_^+^; experimental electronic spectrum of (H_2_O)_3_^+^ on a reduced intensity scale using attenuated laser power; molecular orbitals of (H_2_O)_3_^+^ relevant to the main electronic transitions; calculated relative Gibbs free energies for stable isomers of (H_2_O)_2_^+^; and transition wavelengths, oscillator strengths, and main electronic transitions for each isomer of (H_2_O)_3_^+^ (PDF)
