# Supplementary material for: Observation of the Charge Resonance Band of Hemibonded (H2O)2 + in the Gas Phase
Source: J Phys Chem Lett. 2026 Jun 19;17(26):7322–7. doi: 10.1021/acs.jpclett.6c01649 (PMC13339828; doi:10.1021/acs.jpclett.6c01649)
Supplement: Supplementary file 1 [file jz6c01649_si_001.pdf]

Supporting Information for

Observation of the Charge Resonance Band of Hemibonded $(H_2O)_2^+$ in the Gas Phase

Mizuhiro Kominato,[a] Tatsuki Hosoda,[a] Kenta Mizuse,[b] Susumu Kuma,[c]
Andrey F. Vilesov,[d] and Asuka Fujii*[a]

[a] *Department of Chemistry, Graduate School of Science, Tohoku University, Sendai 980-8578, Japan.*
[b] *Department of Chemistry, School of Science, Kitasato University, Sagamihara, Kanagawa 252-0373, Japan.*
[c] *Department of Physics, College of Science, Rikkyo University, Toshima 171-8501, Japan.*
[d] *Department of Chemistry and Department of Physics and Astronomy, University of Southern California, Los Angeles, California 90089, United States.*

* Corresponding author   email: asuka.fujii.c5@tohoku.ac.jp

Contents

1. **Experimental and computational methods**
2. **Figure S1. Experimental electronic spectrum of $(H_2O)_2^+$ on a reduced intensity scale using attenuated laser power**
3. **Figure S2. Experimental and calculated electronic spectra of $(H_2O)_3^+$**
4. **Figure S3. Experimental electronic spectrum of $(H_2O)_3^+$ on a reduced intensity scale using attenuated laser power**
5. **Figure S4. Molecular orbitals of $(H_2O)_3^+$ relevant to the main electronic transitions**
6. **Table S1. Calculated relative Gibbs free energies (in kJ/mol) for stable isomers of $(H_2O)_2^+$**
7. **Table S2. Transition wavelengths, oscillator strengths, and main electronic transitions for each isomer of $(H_2O)_3^+$**

1. Experimental and computational methods

Details of the experimental apparatus have been described elsewhere.[1] The $(H_2O)_n^+$ (n = 2 and 3) radical cation clusters were generated by electron impact ionization in a supersonic jet of water vapor seeded in Ar gas at a stagnation pressure of 5MPa, which was expanded through a high-pressure pulsed valve (Even–Lavie valve).[2] The produced $(H_2O)_n^+$ cations were mass-selected at the first stage of the quadrupole mass spectrometer and then irradiated with UV-visible (vis) light in the octupole ion guide. When the UV-vis light frequency is resonant with the electronic transition of $(H_2O)_n^+$, fragment ions such as H_2O^+ or H_3O^+ ions are produced. No other fragments were observed, indicating the evaporation of all neutral molecules upon photoexcitation. By monitoring the intensity of mass-selected fragment ions at the second stage of the quadrupole mass spectrometer, UV-vis PD spectra of the $(H_2O)_n^+$ were recorded. For the mass selection of parent ions, the mass resolution was set to approximately $\Delta m/z \leq 1$ to exclude the corresponding protonated clusters, $H^+(H_2O)_n$. While these clusters do not absorb in the observed spectral region and their minimal contamination does not affect the spectra, they were removed to ensure sample purity. In contrast, the mass resolution for the detection of the fragment ions was intentionally reduced to enhance signal intensity. Consequently, both H_2O^+ and H_3O^+ were detected without being distinguished when detecting the H_2O^+/H_3O^+ channel.

The UV-vis light source used was an OPO (NT342, EKSPLA). Although the linewidth of the UV-vis light output from the OPO is less than 5 cm^{-1}, the observed spectra are expected to lack fine structure; therefore, measurements were performed using a step scan with 5 nm increments. To determine the peak positions of the OH moiety absorption, spectra were recorded in 2.5 nm increments (see details in Figures S1 and S3 in the Supporting Information (SI)).

Quantum chemical calculations of $(H_2O)_n^+$ were performed using the Gaussian 16 program package.[3] Geometry optimizations were carried out at the CCSD/aug-cc-pVTZ level. Additionally, excited-state calculations were conducted at the EOM-CCSD/aug-cc-pVTZ level to determine the vertical excitation energies and oscillator strengths of $(H_2O)_n^+$.

References

1. Mizuse, K.; Fujii, A. Infrared Photodissociation Spectroscopy of $H^+(H_2O)_6{\cdot}M_m$ (M = Ne, Ar, Kr, Xe, H_2, N_2, and CH_4): Messenger-Dependent Balance between H_3O^+ and $H_5O_2^+$ Core Isomers. *Phys. Chem. Chem. Phys.* **2011**, 13, 7129–7135.
2. Even, U.; Jortner, J.; Noy, D.; Lavie, N.; Cossart-Magos, C. Cooling of Large Molecules below 1 K and He Clusters Formation. *J. Chem. Phys.* **2000**, 112, 8068–8071.
3. Frisch, M. J.; Trucks, G. W.; Schlegel, H. B.; Scuseria, G. E.; Robb, M. A.; Cheeseman, J. R.; Scalmani, G.; Barone, V.; Petersson, G. A.; Nakatsuji, H.; Li, X.; Caricato, M.; Marenich, A. V.; Bloino, J.; Janesko, B. G.; Gomperts, R.; Mennucci, B.; Hratchian, H. P.; Ortiz, J. V.; Izmaylov, A. F.; Sonnenberg, J. L.; Williams-Young, D.; Ding, F.; Lipparini, F.; Egidi, F.; Goings, J.; Peng, B.; Petrone, A.; Henderson, T.; Ranasinghe, D.; Zakrzewski, V. G.; Gao, J.; Rega, N.; Zheng, G.; Liang, W.; Hada, M.; Ehara, M.;

Toyota, K.; Fukuda, R.; Hasegawa, J.; Ishida, M.; Nakajima, T.; Honda, Y.; Kitao, O.; Nakai, H.; Vreven, T.; Throssell, K.; Montgomery, J. A., Jr.; Peralta, J. E.; Ogliaro, F.; Bearpark, M. J.; Heyd, J. J.; Brothers, E. N.; Kudin, K. N.; Staroverov, V. N.; Keith, T. A.; Kobayashi, R.; Normand, J.; Raghavachari, K.; Rendell, A. P.; Burant, J. C.; Iyengar, S. S.; Tomasi, J.; Cossi, M.; Millam, J. M.; Klene, M.; Adamo, C.; Cammi, R.; Ochterski, J. W.; Martin, R. L.; Morokuma, K.; Farkas, O.; Foresman, J. B.; Fox, D. J. *Gaussian 16*, Revision A.03, Gaussian, Inc., Wallingford, CT, 2016.

2. Figure S1. Experimental electronic spectrum of $(H_2O)_2^+$ on a reduced intensity scale using attenuated laser power

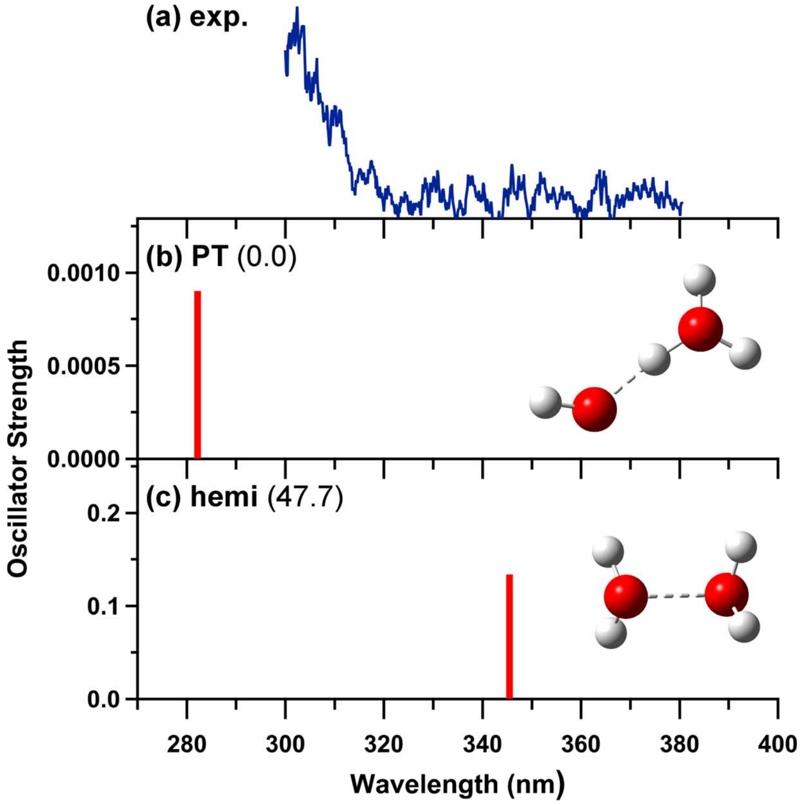

Figure S1. (a) UV-vis photodissociation spectrum of $(H_2O)_2^+$ recorded on a reduced intensity scale using attenuated laser power. The spectrum was measured by monitoring the H_2O^+/H_3O^+ fragment channels. (b-c) Optimized structures and their calculated spectra of (b) the proton-transferred (PT) form and (c) the hemibonded (hemi) form of $(H_2O)_2^+$, respectively. Spectral calculations were performed at the EOM-CCSD/aug-cc-pVTZ level. The values in parentheses are the relative energies (kJ/mol) at the CCSD/aug-cc-pVTZ level, with respect to the most stable PT isomer. Zero-point energy (ZPE) corrections are included.

Figure S1 shows the electronic spectrum of $(H_2O)_2^+$ recorded with attenuated laser power and plotted on a reduced fragment ion intensity scale, in contrast to Figure 1 of the main text. The laser power was also significantly attenuated to avoid saturation of the OH radical band at around 300 nm. Under these conditions, the contribution of the CR band cannot be observed. Note that the laser output is limited to wavelengths above 300 nm.

3. Figure S2. Experimental and calculated electronic spectra of $(H_2O)_3^+$

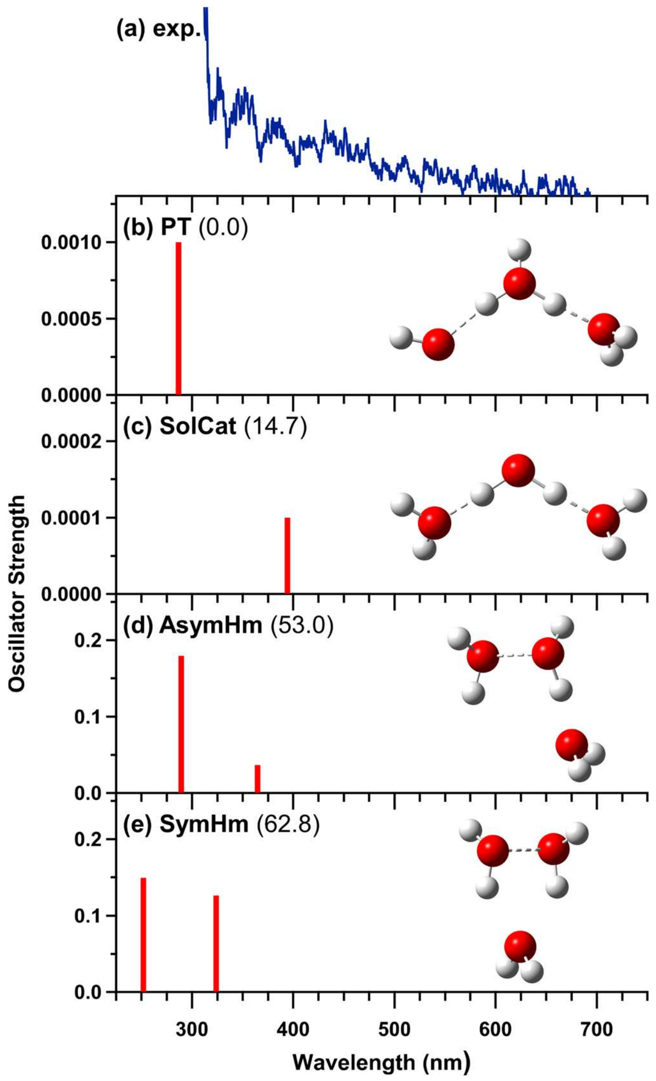

Figure S2. (a) UV-vis photodissociation spectrum of $(H_2O)_3^+$ measured by monitoring the H_2O^+/H_3O^+ fragment channels. (b-e) Optimized structures and their calculated spectra of (b) the proton-transferred (PT) form, (c) the solvated cation (SolCat) form, (s) the asymmetric hemibonded (AsymHm) form, and (e) the symmetric hemibonded (SymHm) form of $(H_2O)_3^+$, respectively. Spectral calculations were performed at the EOM-CCSD/aug-cc-pVTZ level. The values in parentheses are the relative energies (kJ/mol) at the CCSD/aug-cc-pVTZ level, with respect to the most stable PT isomer. Zero-point energy (ZPE) corrections are included.

Figure S2(a) shows the experimental UV-vis photodissociation spectrum of $(H_2O)_3^+$. Similar to the dimer, a broad absorption feature is observed from 600 nm toward shorter wavelengths, overlapped by another distinctly intense band rising sharply from around 320 nm. However, the absorption maximum of the former band could not be clearly identified. The spectrum on a reduced intensity scale (and weaker laser power) is provided in Figure S3, where the absorption maximum of the latter band is located at 305 nm.

The results of the stable structure search for $(H_2O)_3^+$ and the corresponding calculated UV-vis spectra are shown in Figures S2(b)–(e). Four representative structures were identified: (b) the proton-transferred (PT) form, (c) the solvated cation (SolCat) form, where a water radical cation is hydrogen-bonded to a neutral water molecule, and (d, e) the asymmetric (AsymHm) and symmetric (SymHm) forms, in which a hemibonded dimer is solvated by a neutral water molecule. The relative energies (in kJ/mol) of these isomers are shown in parentheses in the figure. Molecular orbitals relevant to the calculated transitions are shown in Figure S4, and the calculated transition wavelengths are summarized in Table S2. The AsymHm and SymHm forms exhibit CR bands of the hemibond at 370 and 320 nm, respectively. These band positions, particularly that of the former, are in good agreement with the experimental observation. However, the SolCat form also shows a band at 395 nm, which provides better agreement in terms of position. Although its transition intensity is approximately 100 times weaker, its relative energy is about 40-50 kJ/mol lower than those for the AsymHm and SymHm forms. Furthermore, in the hemibonded form of $(H_2O)_3^+$, the hemibonded dimer core is hydrogen-bonded to the remaining water molecule. Consequently, isomerization to the most stable PT form via proton transfer occurs more readily compared to $(H_2O)_2^+$, which requires the flipping of one water molecule. Therefore, it is expected that kinetical trapping of the hemibonded form will be more difficult in $(H_2O)_3^+$. Consequently, it remains difficult to unambiguously identify the origin of the weak absorption in the visible region.

The most stable PT form shows an OH-derived absorption at 287 nm, slightly red-shifted from that of $(H_2O)_2^+$, clearly accounting for the relatively intense absorption around 300 nm.

4. Figure S3. Experimental electronic spectrum of $(H_2O)_3^+$ on a reduced intensity scale using attenuated laser power

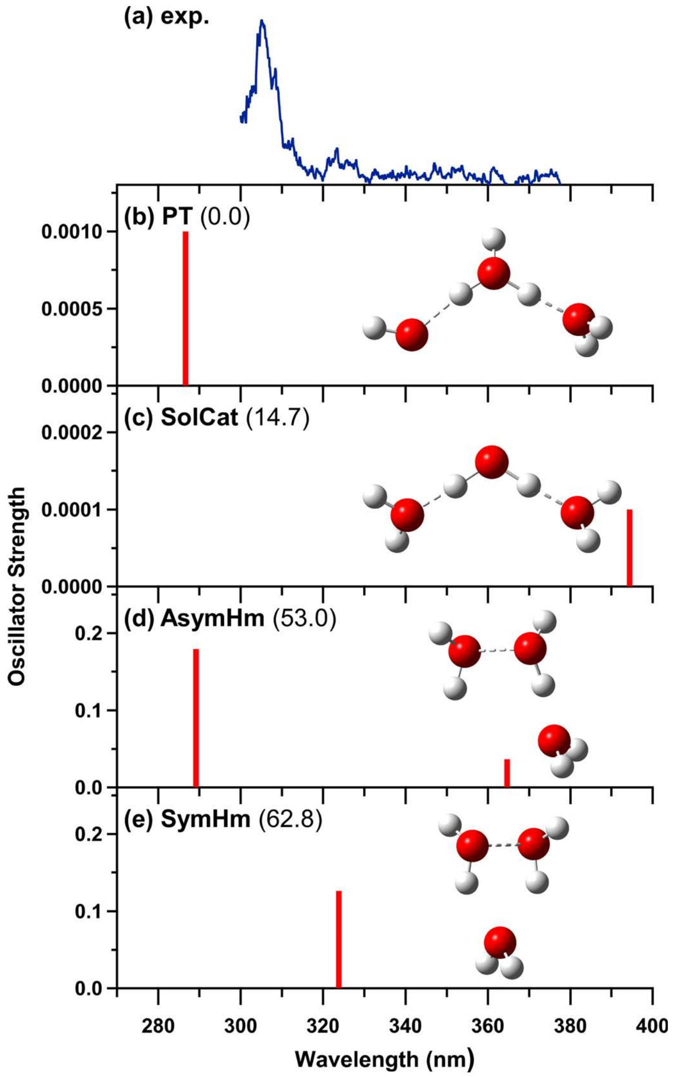

Figure S3. (a) UV-vis photodissociation spectrum of $(H_2O)_3^+$ recorded with attenuated laser power and plotted on a reduced intensity scale. The spectrum was measured by monitoring the H_2O^+/H_3O^+ fragment channels. (b-e) Optimized structures and their calculated spectra of (b) the proton-transferred (PT) form, (c) the solvated cation (SolCat) form, (d) the asymmetric hemibonded (AsymHm) form, and (e) the symmetric hemibonded (SymHm) form of $(H_2O)_3^+$, respectively. Spectral calculations were performed at the EOM-CCSD/aug-cc-pVTZ level. The values in parentheses are the relative energies (kJ/mol) at the CCSD/aug-cc-pVTZ level, with respect to the most stable PT isomer. Zero-point energy (ZPE) corrections are included.

Figure S3 shows the electronic spectrum of $(H_2O)_3^+$ recorded with attenuated laser power and plotted on a reduced fragment ion intensity scale, in contrast to Figure S2. The laser power was also significantly attenuated to avoid saturation of the OH radical band at around 300 nm. Under these conditions, the weak, broad absorption extending from around 300 nm into the visible region observed in Figure S2 is not observed.

5. Figure S4. Molecular orbitals of $(H_2O)_3^+$ relevant to the main electronic transitions

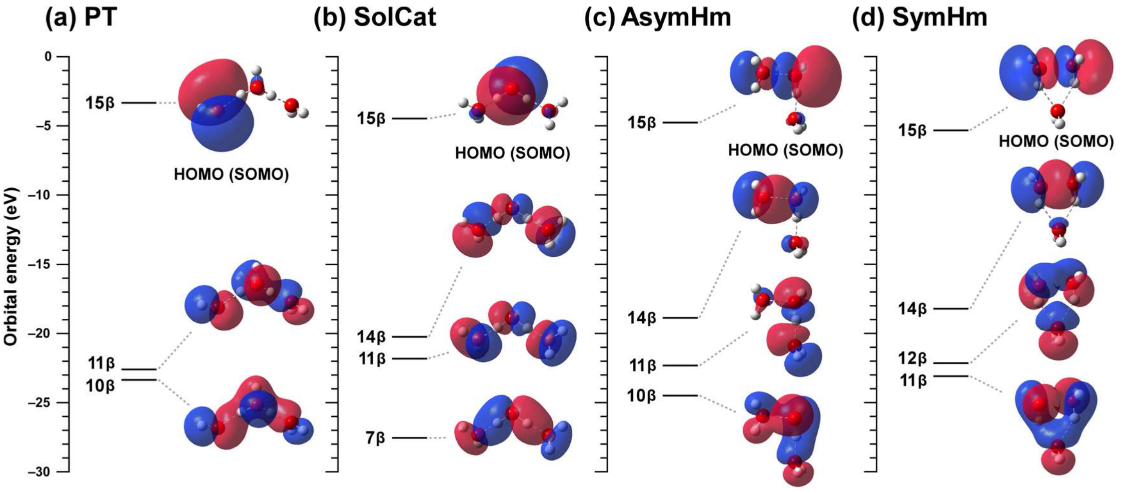

Figure S4. Molecular orbitals of $(H_2O)_3^+$ relevant to the main electronic transitions and their orbital energies (in eV) for (a) the PT form, (b) the SolCat form, (c) the AsymHm form, and (d) the SymHm form. The transition wavelengths and their main transitions are summarized in Table S2. All calculations were performed at the EOM-CCSD/aug-cc-pVTZ level, and the molecular orbitals are plotted with an isovalue of 0.02.

6. Table S1. Calculated relative Gibbs free energies (in kJ/mol) for stable isomers of $(H_2O)_2^+$

Table S1. Calculated relative Gibbs free energies (in kJ/mol) for stable isomers of $(H_2O)_2^+$. All calculations were performed at the CCSD/aug-cc-pVTZ level. Zero point energy (ZPE) corrections are included.

	0 K	100 K	200 K	298K
PT	0	0	0	0
hemi	47.7	48.3	49.0	50.0

7. Table S2. Transition wavelengths, oscillator strengths, and main electronic transitions for each isomer of $(H_2O)_3^+$

Table S2. Transition wavelengths, oscillator strengths, and main electronic transitions for each isomer of $(H_2O)_3^+$. Only transitions with significant excitation amplitudes are listed, and their amplitudes are given in parentheses. All calculations were performed at the EOM-CCSD/aug-cc-pVTZ level.

Isomer	Transition wavelength (nm)	Oscillator strength	Main transitions
PT	286.57	0.0010	10β→15β (0.596)<br>11β→15β (0.604)
SolCat	394.38	0.0001	7β→15β (−0.350)<br>11β→15β (0.539)<br>14β→15β (0.647)
AsymHm	364.69	0.0366	10β→15β (−0.530)<br>11β→15β (−0.433)<br>14β→15β (0.437)
	289.17	0.1795	14β→15β (0.785)
SymHm	323.84	0.1264	11β→15β (0.450)<br>12β→15β (−0.390)<br>14β→15β (−0.723)
	251.71	0.1495	11β→15β (−0.517)<br>12β→15β (0.508)<br>14β→15β (−0.591)
